# Phylogenetic distribution and membrane topology of the LytR-CpsA-Psr protein family

**DOI:** 10.1186/1471-2164-9-617

**Published:** 2008-12-19

**Authors:** Judith Hübscher, Lucas Lüthy, Brigitte Berger-Bächi, Patricia Stutzmann Meier

**Affiliations:** 1 Institute of Medical Microbiology, University of Zürich, Zürich, Switzerland

## Abstract

**Background:**

The bacterial cell wall is the target of many antibiotics and cell envelope constituents are critical to host-pathogen interactions. To combat resistance development and virulence, a detailed knowledge of the individual factors involved is essential. Members of the LytR-CpsA-Psr family of cell envelope-associated attenuators are relevant for β-lactam resistance, biofilm formation, and stress tolerance, and they are suggested to play a role in cell wall maintenance. However, their precise function is still unknown. This study addresses the occurrence as well as sequence-based characteristics of the LytR-CpsA-Psr proteins.

**Results:**

A comprehensive list of LytR-CpsA-Psr proteins was established, and their phylogenetic distribution and clustering into subgroups was determined. LytR-CpsA-Psr proteins were present in all Gram-positive organisms, except for the cell wall-deficient *Mollicutes *and one strain of the *Clostridiales*. In contrast, the majority of Gram-negatives did not contain LytR-CpsA-Psr family members. Despite high sequence divergence, the LytR-CpsA-Psr domains of different subclusters shared a highly similar, predicted mixed a/β-structure, and conserved charged residues. PhoA fusion experiments, using MsrR of *Staphylococcus aureus*, confirmed membrane topology predictions and extracellular location of its LytR-CpsA-Psr domain.

**Conclusion:**

The LytR-CpsA-Psr domain is unique to bacteria. The presence of diverse subgroups within the LytR-CpsA-Psr family might indicate functional differences, and could explain variations in phenotypes of respective mutants reported. The identified conserved structural elements and amino acids are likely to be important for the function of the domain and will help to guide future studies of the LytR-CpsA-Psr proteins.

## Background

The cell envelope forms a protective shield around bacteria and is also the site of primary host-pathogen interactions. Its composition and surface characteristics are therefore important in pathogenesis, and may, in view of increasing resistance against all commonly used cell wall-directed antibiotics, present novel potential antibacterial targets.

The LytR-CpsA-Psr family of cell envelope-associated transcriptional attenuators has been brought into focus of scientific interest upon the discovery that members of this family influence various virulence factors as well as antibiotic resistance of important human pathogens and, interestingly, seem to play a role in bacterial cell envelope maintenance [1-5]. Therefore, this protein family represents a promising target to gain more insight into virulence and antibiotic resistance development.

The LytR-CpsA-Psr family members are putative transmembrane proteins carrying a so-called LytR-CpsA-Psr domain, which is predicted to be extracellular. LytR was first described in *Bacillus subtilis*, where it acts as an attenuator of the expression of both itself and the divergently transcribed *lytABC *operon, which encodes a putative lipoprotein (LytA), an *N*-acetylmuramoyl-L-alanine amidase (LytC) and its modifier LytB [3]. CpsA is suggested to play a role in transcription activation of the capsular polysaccharide synthesis operon of *Streptococcus agalactiae *[2]. Psr was initially proposed to be a repressor of penicillin-binding protein 5 (PBP5) synthesis in *Enterococcus hirae *[6], however, Sapunaric et al. could neither confirm an effect on PBP5 synthesis nor autolysis nor β-lactam resistance [7]. In contrast, the LytR homolog BrpA affects autolytic activity of *Streptococcus mutans *[1] and positively influences biofilm formation ability as well as acidic and oxidative stress tolerance [5]. Furthermore, BrpA inactivation alters phagocytosis by human polymorphonuclear leukocytes and the outcome of bacteremia in a rat model [8]. In *Staphylococcus aureus*, the LytR-CpsA-Psr member MsrR contributes to β-lactam resistance [4]. MsrR of *S. aureus *as well as Psr of *Enterococcus faecalis *were both shown to increase virulence in the model host *Caenorhabditis elegans *[9,10]. Furthermore, expression of SA0908, another member of the LytR-CpsA-Psr family in *S. aureus*, is increased during infection in a murine renal abscess model [11].

The function of the LytR-CpsA-Psr domain, however, is still unknown and so far information about these proteins is based on phenotypic characterizations. A more comprehensive knowledge about the occurrence of LytR-CpsA-Psr proteins and analyses of their sequence will provide a basis for experimental determination of interactions or structure/function relationships.

In the present study, we investigated the phylogenetic distribution of the LytR-CpsA-Psr proteins and analyzed secondary structure predictions. In addition, the staphylococcal LytR-CpsA-Psr protein MsrR was used as a model to confirm membrane topology.

## Results and discussion

### Sequence collection of LytR-CpsA-Psr proteins

A comprehensive sequence collection was obtained by searching the InterPro database [12] for LytR-CpsA-Psr members (InterPro entry IPR004474) and using PSI-BLAST [13] to identify further homologous sequences as described in the Methods section. Sequences representing fragments of the LytR-CpsA-Psr domain were excluded from the studies. The revised dataset comprised 1'079 sequences.

### Occurrence of the LytR-CpsA-Psr domain

**Figure 1:**
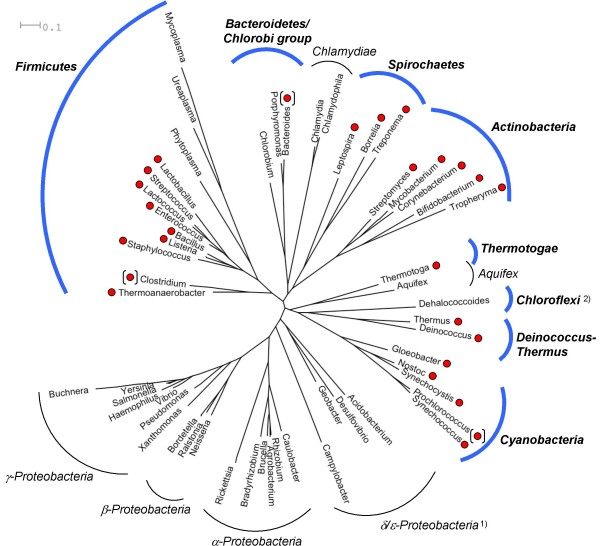
**Overview of the occurrence of LytR-CpsA-Psr family proteins in bacteria**. The phylogenetic tree is based on the interactive tree of life (iTOL) [65], which was pruned to a selection of genera representing the different phyla. The eight phyla, where LytR-CpsA-Psr family proteins were found, are printed in bold, and red circles indicate their presence in the respective genera. In case not all members of a certain genus harbor LytR-CpsA-Psr proteins, the circle was put in brackets. For a comprehensive description of the occurrence of LytR-CpsA-Psr domain refer to the text. ^1)^A single fragment of a LytR-CpsA-Psr protein was detected in the δ-*Proteobacterium Plesiocystis pacifica *strain SIR-1. ^2)^Except for the *Dehalococcoides*, which are representing the phylum of the *Chloroflexi *in this tree, all members of the *Chloroflexi *contain LytR-CpsA-Psr family members.

Members of the LytR-CpsA-Psr family were found in the eight bacterial phyla *Actinobacteria*, *Bacteroidetes*, *Chloroflexi*, *Cyanobacteria*, *Deinococcus-Thermus*, *Firmicutes*, *Spirochaetes*, and *Thermotogae *(Figure 1). In addition, a LytR-CpsA-Psr fragment was identified in *Plesiocystis pacifica *strain SIR-1, which is a member of the *δ-Proteobacteria*.

The LytR-CpsA-Psr domain is restricted to the kingdom of bacteria with two exceptions occurring in the moss *Physcomitrella patens *subsp. *patens *(*Bryophyta*, Moss Superclass V) and the freshwater amoeba *Paulinella chromatophora*. *Physcomitrella *is widely used as a model organism in plant genetics as it undergoes integrative homologous recombination with a high efficiency [14], while *Paulinella *contains photosynthetic inclusions (chromatophores) affiliated with the cyanobacteria *Prochlorococcus *and *Synechococcus *spp. [15]. BLAST analysis of the LytR-CpsA-Psr sequences found in *Paulinella *and *Physcomitrella *revealed high similarities to those found in *Synechococcus *sp. WH5701 and *Paenibacillus *sp. JDR-2, respectively (data not shown). It is therefore conceivable that the LytR-CpsA-Psr proteins found in *Physcomitrella *and *Paulinella *are of bacterial origin.

Analysis of the genomes available at the National Center for Biotechnology Information (NCBI) [16] revealed that all of the completely sequenced *Firmicutes *contain at least one member of the LytR-CpsA-Psr family, except for *Clostridium kluyveri *strain DSM 555 and the whole order of *Mollicutes*. In the phylum of *Actinobacteria*, LytR-CpsA-Psr proteins were found in every completely sequenced genome and the same was true for *Deinococcus-Thermus*, *Spirochaetes*, and *Thermotogae*. In contrast, only two strains of the *Bacteroidetes*, i.e. *Pedobacter *sp. BAL39 (*Sphingobacteriales*) and *Bacteroides capillosus *ATCC 29799 (*Bacteroidales*) harbor LytR-CpsA-Psr proteins, but none of the completely sequenced strains. Among the *Chloroflexi*, the *Dehalococcoides *do not possess LytR-CpsA-Psr members, whereas they were found in the orders *Chloroflexales *and *Herpetosiphonales*. Most of the *Cyanobacteria *genomes contain LytR-CpsA-Psr members, but only two out of twelve *Prochlorococcus marinus *genomes.

#### Characteristics of LytR-CpsA-Psr carriers

The organisms having LytR-CpsA-Psr proteins represent the immense diversity of bacteria with regard to morphology, ultrastructure, motility, metabolic characteristics, and habitat preferences. LytR-CpsA-Psr family members were found in pathogens of human, animals, or plants as well as in non-pathogenic organisms, in generalists and specialists such as hyperthermophilic bacteria. This heterogeneity raises the question: Is there a common trait to the LytR-CpsA-Psr carriers? One of the most striking observations was that the LytR-CpsA-Psr proteins are absent in the large Gram-negative phylum of *Proteobacteria *and many other Gram-negative groups, while all of the Gram-positive bacteria contain at least one copy. Given the proposed role of the LytR-CpsA-Psr proteins in cell envelope maintenance and the lack of the respective domain in the cell wall-deficient order *Mollicutes*, this finding might indicate a correlation with the Gram-positive cell wall in particular. However, the list of LytR-CpsA-Psr carriers also comprised *Spirochaetes *and numerous organisms classified as Gram-negatives, such as members of the *Cyanobacteria*, *Thermus*, *Thermotogae*, *Bacteroidetes*, and *Chloroflexi*.

Nevertheless, many of the Gram-negative LytR-CpsA-Psr carriers show features of a typical Gram-positive cell wall, such as a thick, multilayered peptidoglycan, a high cross-linking extent, or polysaccharides cross-linked to the peptidoglycan. Intermediate types between a typical Gram-negative and Gram-positive cell wall are for example observed in various *Cyanobacteria *[17], *Chloroflexus aurantiacus *[18], and *Deinococcus radiodurans *[19]. In conclusion, however, a common feature related to peptidoglycan composition or amount was not apparent.

#### Loss and gain of LytR-CpsA-Psr proteins in the course of evolution

The LytR-CpsA-Psr proteins described so far are not essential, and viable knock out mutants were obtained in *S. aureus*, *S. mutans*, and *E. hirae *[1,4,7]. These particular organisms, however, contain more than one member of the LytR-CpsA-Psr family. It would be interesting to know if the inactivation of all LytR-CpsA-Psr proteins affects viability. On the other hand, our sequence collection showed that this protein family was lost in certain organisms in the course of evolution. As mentioned above, only two *Prochlorococcus *strains carry LytR-CpsA-Psr members. The respective strains, MIT9303 and MIT9313, are distinct from other *Prochlorococcus *isolates in that the size and GC content of their genomes are more similar to those of the genus *Synechococcus *[20]. The differences in genome size reflect gene gain and loss during the evolution of *Prochlorococcus*, which lost the LytR-CpsA-Psr proteins after divergence from the MIT9303/MIT9313 clade. Interestingly, besides the LytR-CpsA-Psr members, numerous genes that are present in MIT9303/MIT9313 and most *Synechococcus*, but absent in the other completely sequenced *Prochlorococcus*, are involved in cell envelope biogenesis [20]. Apart from *Prochlorococcus*, *C. kluyveri *is likely to be another example for the loss of LytR-CpsA-Psr proteins. *C. kluyveri *differs from other members of the genus *Clostridium *by unique metabolic features and its genome shows only very low syntheny on protein level to other *Clostridia *[21].

The occurrence of the LytR-CpsA-Psr family in the *Thermotogae*, which are considered an evolutionary early branch of bacteria [22], might indicate that this family already appeared in a common procaryotic ancestor and was lost by some lineages during the process of diversification, e.g. in the *Proteobacteria*. From this point of view, the incidence in *P. pacifica *SIR-1 could be the result of lateral gene transfer. However, the GC content of the LytR-CpsA-Psr protein encoding gene and the GC content of *P. pacifica *SIR-1 do not differ and a more thorough investigation would be required to address this question. Yet, lateral gene transfer plays a significant role in evolution [23], and it is possible that LytR-CpsA-Psr proteins were (re-) acquired in the time course of procaryotic diversification.

### Cluster analysis

Our sequence collection revealed that from one, e.g. in *C. tetani*, to eleven, e.g. in *Streptomyces coelicolor*, any number of LytR-CpsA-Psr members could be found in a single species. In order to investigate if there existed sub-families and a particular distribution, we performed a cluster analysis based on pair-wise sequence similarities resulting from a BLAST all against all search using CLANS [24] with a P-value cut off of 10^-35^.

**Figure 2:**
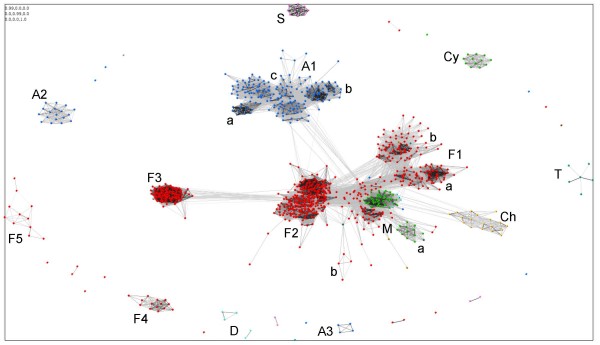
**Two-dimensional CLANS clustering of the LytR-CpsA-Psr proteins**. Proteins are represented by dots and colored according to their taxonomic relationship: blue, *Actinobacteria*; olive, *Bacteroidetes*; green, *Cyanobacteria*; yellow, *Chloroflexi*; turquoise, *Deinococcus-Thermus*; red, *Firmicutes*; dark green, *Physcomitrella*; brown, *Plesiocystis*; pink, *Spirochaetes*; petrol, *Thermotogae*. BLAST sequence similarities are indicated by lines shaded from light grey (P-values < 10^-35^) to black (P-values < 10^-200^). Clusters are indicated using the first character(s) of the corresponding phylum and are numbered in case there is more than one cluster of the same phylum. M denotes the taxonomically mixed 'central' cluster. Subclusters within A1, F1, and M are marked with small letters.

The results mainly reflected taxonomic relationships. Accordingly, the clusters were termed using the first character(s) of the respective phylum followed by consecutive numbers in case of multiple clusters. The majority of sequences grouped in six clusters, i.e. A1 (*Actinobacteria *cluster 1), Ch (*Chloroflexi *cluster), F1–F3 (*Firmicutes *clusters 1 to 3), and M (taxonomically mixed cluster) (Figure 2 and Additional file 1). Besides these main clusters, seven 'peripheric' clusters containing four to 18 proteins were observed: A2, A3, Cy (*Cyanobacteria *cluster), F4, F5, S (*Spirochaetes *cluster), and T (*Thermotogae *cluster). In addition, five sequences of the *Deinococcus-Thermus *group that were found in close proximity to each other were considered an additional cluster D. A small number of proteins did not cluster with any other of the LytR-CpsA-Psr members.

A step-wise increase in the P-value cut off caused the clusters A1, Ch, F1, F2, and M to merge in a supercluster (data not shown), suggesting a close relationship of the enclosed sequences. Decreasing the P-value cut off resulted in disconnection of the clusters into subclusters mainly following phylogenetic affiliations (data not shown). To verify whether unequal family sizes or redundant sequences distorted cluster analysis, CLANS was subsequently applied to a reduced dataset of 576 sequences with a maximum similarity of 90%. The use of this reduced dataset had no significant effect on the outcome of the clustering (data not shown) and the following discussion is based on the results obtained using the unfiltered list of LytR-CpsA-Psr proteins.

#### Cluster M – taxonomically mixed central cluster

Cluster M was the only one containing proteins of organisms belonging to various phyla, i.e. *Firmicutes*, *Actinobacteria*, *Bacteroidetes*, *Deinococcus-Thermus*, and *Cyanobacteria*, and might represent those sequences that have diverged the least from a presumed ancestral LytR-CpsA-Psr protein. A clearly separated subcluster (M_a_) contained exclusively sequences of *Prochlorococcus *and *Synechococcus *species as well as of the amoeba *Paulinella*.

#### Cluster A1 – common Actinobacteria sequences

Most LytR-CpsA-Psr proteins of the high GC Gram-positive organisms grouped in cluster A1. A1-type sequences were identified in each member of the *Actinobacteria *and therefore represent the sequences characteristic for this phylum. The proteins of this cluster were predominantly predicted to have a single transmembrane segment with an extracellular LytR-CpsA-Psr domain, a trait attributed to the majority of LytR-CpsA-Psr proteins.

Within cluster A1, three separated subclusters were distinguished. Subcluster A1_a _enclosed solely sequences of different *Mycobacterium *species, whereas A1_b _and A1_c _both covered sequences of various *Actinobacteria*.

Cluster A1 also harboured FrnA [UniProt:O68907], which contains an SBP_bac5 domain (bacterial extracellular solute-binding protein family 5 domain; PFAM:PF00496). The family 5 solute-binding proteins comprise peptide-binding proteins, such as the oligopeptide transporter subunit OppA of Gram-negative bacteria. FrnA was exclusively found in *Streptomyces roseofulvus*, and is encoded by the first gene of a 25 kb-region, containing genes involved in biosynthesis of the polyketide antibiotic frenolicin [25]. In *Saccharopolyspora erythraea*, two A1-type proteins, SACE_6482 and SACE_6483, are encoded adjacent to *aveBII *(SACE_6480), a dTDP-glucose 4,6-dehydratase involved in the biosynthesis of a polyketide sugar unit precursor and hence of the macrolide antibiotic erythromycin A [26]. However, any involvement of LytR-CpsA-Psr proteins in biosynthesis of secondary metabolites by organisms of the order *Actinomycetales *remains open.

#### Cluster A2 – Actinobacteria proteins with multiple transmembrane segments

A few LytR-CpsA-Psr family members of the *Actinobacteria *clustered in A2. In contrast to the A1-type proteins and the LytR-CpsA-Psr family members in general, those proteins found in A2 were calculated to cross the membrane two to four times. In accordance with predictions for LytR-CpsA-Psr members with one transmembrane helix and independently of the number of predicted transmembrane helices, the LytR-CpsA-Psr domains of A2-type proteins were mainly suggested to be extracellular by the various programs used. A2-type proteins were only present in some organisms of the *Propionibacterineae*, *Streptosporangineae*, and *Micrococcineae*.

#### Cluster A3 – Streptomyces-specific proteins

This cluster contained four sequences, which are rich in glycine and the polar amino acid glutamine predominantly located within a stretched, uncharged segment close to the N-terminus. The A3-type proteins also differ from other LytR-CpsA-Psr members by their exceptional length ranging from 559 to 617 aa and were only found in *Streptomyces*.

#### Cluster F1 – Psr/MsrR-like proteins of Firmicutes

Apart from those sequences comprised in cluster M, the *Firmicutes *sequences divided into three main clusters F1–F3 and two peripheric clusters F4 and F5. Cluster F1 contained proteins of the orders *Bacillales *and *Lactobacillales *(subcluster F1_a_) as well as of *Clostridiales *and the two non-*Firmicutes Pedobacter *sp. (phylum *Bacteroidetes*) and *Fervidobacterium nodosum *(phylum *Thermotogae*) (subcluster F1_b_).

F1-type proteins included Psr of enterococci, which is suggested to be an activator of its own transcription, especially in the presence of ampicillin [27]. In the food-borne pathogen *Listeria monocytogenes*, transcription of *psr *is increased in response to growth at 10°C [28]. Cluster F1 also included MsrR of *Staphylococcus aureus*, which is involved in β-lactam resistance [4]. *msrR *belongs to the cell wall stress stimulon, a set of genes collectively induced upon exposure to antibiotics damaging the cell wall [29,30].

#### Cluster F2 – LytR/BrpA-like proteins of Firmicutes

Cluster F2 mainly contained sequences of the orders *Bacillales *and *Lactobacillales *and included LytR of *B. subtilis *and BrpA of *S. mutans*. BrpA has an impact on autolysis, biofilm formation, stress tolerance, and virulence [1,5], and LytR acts as an attenuator of the *lytABC *operon encoding the major autolytic amidase of *B. subtilis *[3]. LytR belongs to the σ^X ^regulon, which controls genes participating in cell envelope metabolism and modulation [31,32]. YwtF, a further F2-type protein in *B. subtilis*, is a member of the σ^M ^regulon. The σ^M ^regulon plays a role in response to cell envelope stress, and in contrast to *lytR*, *ywtF *gene expression is upregulated by bacitracin and vancomycin [33-35]. A similar situation as in *B. subtilis *was observed for transcription of *lytR *and *ywtF *in *B. licheniformis *[36]. The two F2-type LytR-CpsA-Psr members of *S. aureus*, SA0908 and SA2103 in strain N315, both belong to the cell wall stress stimulon as does *msrR *of cluster F1 [30,37,38].

In *Bacillus thuringiensis *subsp. *thuringiensis *strain 407-1, the F2-type LytR-CpsA-Psr protein EcfY is thought to be encoded in an operon with *sigW *(similar to extracytoplasmic function σ factor) and *ecfX *(putative anti-σ factor). *sigW *and *ecfX *contribute to β-exotoxin production, and *ecfY *was proposed to be involved in negative control of *sigW *expression [39].

In cluster F2, a LytR-CpsA-Psr protein with a type 2 phosphatidic acid phosphatase superfamily domain (PAP2, PFAM:PF01569) was contained, which is encoded by *E. faecalis *EF_3245. Certain PAP2 family members have undecaprenyl pyrophosphate phosphatase activity, and thus may play a role in peptidoglycan biosynthesis by providing the lipid carrier undecaprenyl phosphate [40,41]. Moreover, the LytR-CpsA-Psr protein of the eukaryote *Physcomitrella *also clustered in F2.

The staphylococcal protein LytR (SA0251 in strain N315), the response regulator of the two-component system LytSR [42], does not belong to the LytR-CpsA-Psr protein family despite its name.

#### Cluster F3 – CpsA-like Firmicutes proteins with multiple transmembrane segments

The third *Firmicutes *cluster was formed by the CpsA-like proteins, which are predominantly found in the genus *Streptococcus*. In streptococci, *cpsA *(also *cpsX*, *epsA*, *wzg*) is the first gene of the capsular biosynthesis locus, and CpsA is assumed to be a transcriptional regulator of capsule production in *S. agalactiae *[2]. In *S. pneumoniae*, however, although encapsulation is reduced in a *cpsA *mutant, no evidence for an effect of CpsA on transcription of the *cps *operon was found [43]. Yet, the mutant exhibited reduced tyrosine phosphorylation of CpsD, which is thought to be required for production of elevated amounts of capsule [43].

All F3-type proteins were predicted to have three transmembrane helices with an intracellular N-terminus. Additionally, CpsA and related proteins of streptococci possess a DNA polymerase processivity factor domain (DNA-PPF, PFAM:PF02916), a subdomain of the replisome sliding clamp unit. However, the subunits of the sliding clamp ring are composed of two homologous subdomains, the DNA-PPF domain and a gp45-slide C subdomain (PFAM:PF09116) [44], which is absent in CpsA. Moreover, in CpsA the DNA-PPF domain was predicted to span the third putative membrane segment with the major portion of the domain located extracellularly. These findings imply that the DNA-PPF domain found in CpsA proteins fulfils a different function. The extracellular portion of the CpsA DNA-PPF domain overlaps with a structural periplasmic binding protein-like II domain (SCOP superfamily 53850) as identified using SCOP (Structural Classification Of Proteins) [45]. This superfamily includes various binding proteins such as phosphate-binding proteins, glutamine-binding proteins, or ferric-binding proteins. Interestingly, SCOP superfamily 53850 also covers the family 5 solute-binding proteins discussed with respect to the A1-type LytR-CpsA-Psr protein FrnA.

A number of proteins of the *Clostridiales *also clustered in F3. Although these proteins did not produce a significant hit to the DNA-PPF domain in a PFAM search, they were of similar length as CpsA (456–559 amino acids), and four of them [UniProt:A5KKW2, A8RE01, A8R906, and B1C5F5) were also predicted to carry a periplasmic binding protein-like II domain.

#### Clusters F4 and F5 – small 'peripheric' Firmicutes subclusters

Cluster F4 only contained sequences of *Lactococcus lactis *and *Streptococcus thermophilus*, whereas F5 only contained sequences of certain *Clostridiales*. The F4- and F5-type sequences showed the same transmembrane organization, and family domain architecture as those of the *Firmicutes *main clusters F1 and F2.

#### Clusters Ch, Cy, D, S and T – other taxonomy-specific clusters

The sequences of the *Chloroflexi *grouped in one single cluster (Ch) closely related to M, and all LytR-CpsA-Psr members of *Thermotoga *and one of the two members found in *F. nodosum *assembled in the *Thermotogae *cluster T. Cluster D comprised those *Deinococcus-Thermus *sequences not included in cluster M. Cluster Cy enclosed a set of *Synechococcus *and *Prochlorococcus *sequences, and finally the LytR-CpsA-Psr members of the *Spirochaetes Borrelia *were grouped in cluster S. The sequences of the other *Spirochaetes*, i.e. *Leptospira *and *Treponema*, were not included in any cluster.

All sequences enclosed in these clusters showed for the LytR-CpsA-Psr proteins a characteristic organization with a short cytoplasmic domain, one transmembrane segment, and a putative extracellular domain carrying the LytR-CpsA-Psr element.

### Phylogenetic distribution

**Figure 3:**
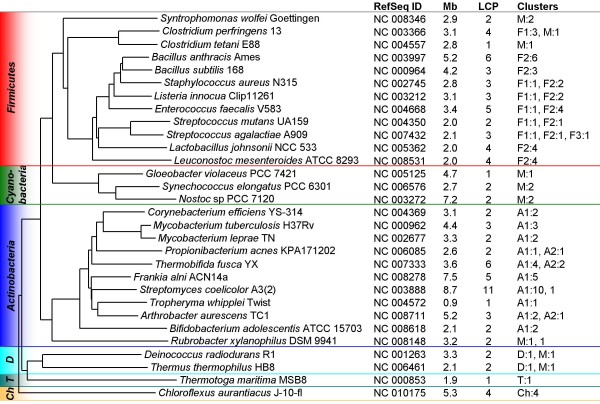
**Distribution of LytR-CpsA-Psr proteins in selected genomes**. The distribution of the LytR-CpsA-Psr family members was investigated in 30 fully sequenced bacterial strains. Phylogenetic relationships are shown by means of a species tree based on the 16S rRNA gene sequences. The NCBI RefSeq database accession numbers and the sizes of the genomes (total of all DNA molecules) in mega bases (Mb) are indicated. The number of LytR-CpsA-Psr family members (LCP) identified in the corresponding genome are given and the last column summarizes in which CLANS cluster they were found as follows: 'denotation of the cluster':'number of proteins'. A number without an indication of a cluster means that this sequence did not group with any other sequences.

For a more detailed analysis of the phylogenetic distribution of the LytR-CpsA-Psr proteins, 30 fully sequenced strains representing the six phyla *Actinobacteria*, *Cyanobacteria*, *Chloroflexi*, *Deinococcus-Thermus*, *Firmicutes*, and *Thermotogae *were selected (Additional file 2). Figure 3 summarizes the number of LytR-CpsA-Psr proteins detected in these strains and the results of the CLANS clustering related to the species tree, which was constructed based on the 16S rRNA gene sequences.

**Figure 4:**
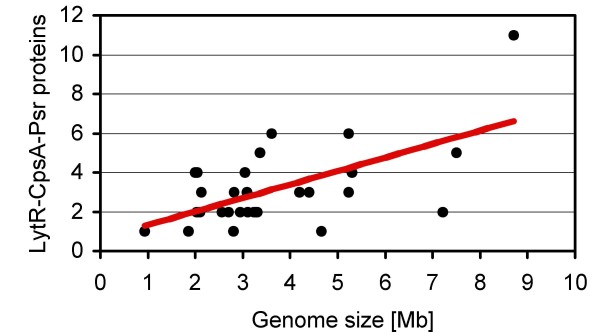
**Relationship of the genome size to the number of LytR-CpsA-Psr proteins**. With an increasing genome size generally an increased number of LytR-CpsA-Psr family members was found in the 30 strains investigated. The coefficient of correlation was 0.621, indicating an intermediate positive correlation, which is illustrated by the linear regression line (red).

Comparison of the genome sizes and the number of LytR-CpsA-Psr members in the respective organisms revealed a positive correlation (Figure 4). There were, however, exceptions like *Nostoc *sp. PCC 7120 with a genome size of 7.2 Mb but only two LytR-CpsA-Psr proteins.

The accumulation of multiple LytR-CpsA-Psr proteins most probably often happened through duplication from an ancestral species- or group-specific sequence, leading to as many as 11 members in *S. coelicolor*. Ten of the *S. coelicolor *LytR-CpsA-Psr proteins clustered in A1, while the eleventh [UniProt:Q9RDK8] clustered in A3. In contrast to Q9RDK8, all genes of the ten A1-type proteins lie within the central core region of the *S. coelicolor *genome, and seven of the LytR-CpsA-Psr genes are consecutively encoded. The core region of *S. coelicolor *comprises genes likely to be essential, such as genes involved in cell division, DNA replication, transcription, translation, and amino acid biosynthesis [46].

Gene duplication might also have happened in an ancestor of the *Deinococcus-Thermus *group, which is likely to have carried two members of the LytR-CpsA-Psr family: one of the M cluster-type and a more differentiated, phylum-specific one. Both of these LytR-CpsA-Psr proteins have been retained in all of the fully sequenced organisms of this taxon.

An identical distribution of LytR-CpsA-Psr proteins was found in the genus *Staphylococcus *and *Listeria*, which share one member in the MsrR/Psr-like cluster F1 and two members in the LytR/BrpA-like cluster F2. Only *Staphylococcus haemolyticus *encodes a fourth LytR-CpsA-Psr protein, CapM, which clusters in F2 and is the last gene of a putative capsular polysaccharide biosynthesis locus *capA*-*capM*. *capA*-*capG *show ≥ 76% amino acid identity to the *cap5[8]A*-*cap5[8]G *genes of *S. aureus*, while *capH*-*capM *are unique to *S. haemolyticus *and might be of exogenous origin [47]. The three LytR-CpsA-Psr proteins common to staphylococci and listeria most likely were present in an evolutionary older organism before their phylogenetic separation, however, the cluster analysis leaves the phylogeny of the LytR-CpsA-Psr proteins in general open, and a hypothesis that cluster M represents the most original group remains speculative.

In order to infer evolutionary relatedness of the LytR-CpsA-Psr proteins represented by the 30 strains selected, phylogenetic trees were constructed. However, the LytR-CpsA-Psr proteins show only low amino acid identity and the domain characterizing this family is only about 150 amino acids in length (average value of the respective 93 sequences), rendering construction of phylogenetic trees problematic. Reliable bootstrap support was only obtained close to the terminal nodes, though the results of the cluster analyses were supported (Additional file 3), reflecting taxonomic relationships as shown in the 16S rRNA gene tree in Figure 3.

### Secondary structure prediction

A subset of twelve sequences representing the LytR-CpsA-Psr proteins of clusters M, A1, F1, F2, F3, Ch, and T was subjected to secondary structure prediction. The sequences analyzed included the three eponymous proteins LytR, CpsA, and Psr, as well as other family members that have been described in literature, i.e. BrpA, MsrR, and SA0908. The sequences varied in length from 293 to 485 amino acids and they showed very different contents of charged amino acids and theoretical isoelectric points (pI) ranging from 5.17 to 9.66 (Additional file 4). All sequences contained one consensus transmembrane region predicted by all of the four servers used, except for CpsA, which was predicted to have three transmembrane helices. The majority of the membrane topology predictions suggested an intracellular N-terminus, implicating an extracellular location of the LytR-CpsA-Psr domain.

**Figure 5:**
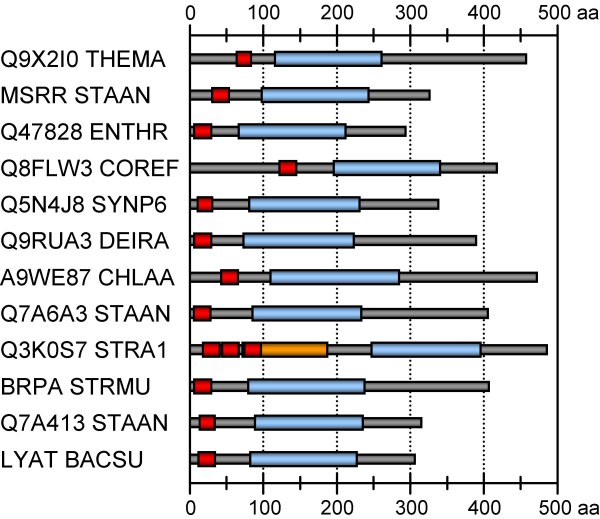
**Architectures of the LytR-CpsA-Psr proteins used for secondary structure prediction**. Most of the sequences showed a for LytR-CpsA-Psr proteins typical domain organization with a short N-terminal tail, a single transmembrane domain (red), and a long C-terminal part carrying the LytR-CpsA-Psr domain (light blue). An exception of this architecture is found in the group of CpsA-like proteins, represented by CpsX of *S. agalactiae *(Q3K0S7_STRA1), which contain three predicted transmembrane helices and a DNA polymerase processivity factor domain (orange) in addition to the LytR-CpsA-Psr domain. The sequence identifiers correspond to the UniProt entry names (see Additional file 4).

The putative cytoplasmic tails of eight sequences were very short ranging from only six to 19 amino acids while CE2746 of *C. efficiens *[UniProt:Q8FLW3] showed a long cytoplasmic domain of 121–123 amino acids. A graphical overview of the respective architectures, illustrating also variations in the length of the extracellular domains, is given in Figure 5. The overall identities of the amino acid sequences were only between 17 and 39% (Additional file 5).

**Figure 6:**
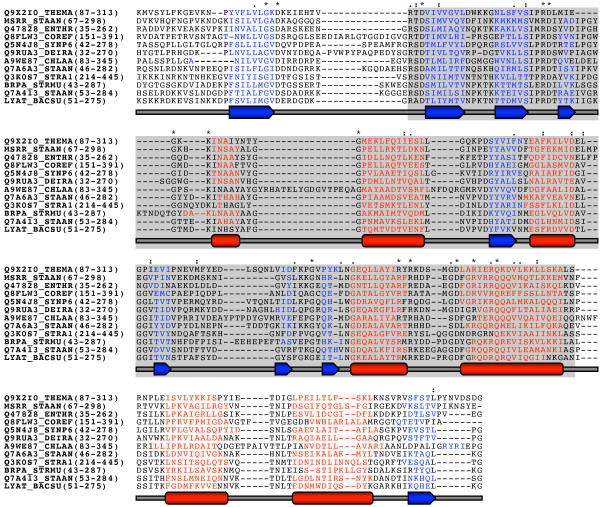
**Secondary structure prediction of the extended LytR-CpsA-Psr domain**. Full length sequences were aligned using MUSCLE and secondary structures were predicted using the prediction servers SsPro, PSIPRED, and Jpred. The consensus α-helices (red) and β-sheets (blue) based on the results of the three servers are shown for the extended LytR-CpsA-Psr domain. A schematic drawing underneath the aligned sequences further illustrates the sequential order of predicted α-helices (barrels) and β-sheets (arrows). The LytR-CpsA-Psr domain as defined in the PFAM database (PF03816) is shaded in grey. Fully conserved residues are marked by an asterisk (*) above the aligned sequences. A dash (-) represents a gap in the alignment. A colon (:) indicates that one of the following groups is conserved: STA, MILV, MILF, or NDEQ; a dot (.) indicates that one of the following groups is conserved: FVLIM, NEQHRK, SNDEQK, or SAG. The sequence identifiers correspond to the UniProt entry names (see Additional file 4), and the amino acid range covering the extended LytR-CpsA-Psr domain of each sequence is given in parentheses.

There was a good overlap of the predicted structural elements within the LytR-CpsA-Psr domain and its flanking regions (extended LytR-CpsA-Psr domain), revealing a mixed a/β-structure (Figure 6). Secondary structure predictions have to be interpreted with caution and only experimental resolution will allow accurate interpretation of structure-function relationships. However, preliminary circular dichroism (CD) spectra of the purified and refolded extracellular domain of MsrR of *S. aureus *support predicted α-helix and β-sheet contents (L. Lüthy, unpublished).

The amino acid identities of the extended LytR-CpsA-Psr domains ranged from 21 to 44% (Additional file 5) and were hence only little above those of the full length proteins. Interestingly, the few fully conserved residues included nine charged amino acids: aspartic acid, arginine, and lysine. Charged residues are often involved in the active site of proteins, where they interact with charged ligands or bind substrates. Moreover, they may play a role in formation of protein-protein complexes and are important for folding, stability, and solubilization of proteins [48]. It is therefore very likely that at least some of the conserved residues are indispensable for the function of the LytR-CpsA-Psr domain in these proteins.

High sequence divergence was observed with regard to some of the peripheric clusters, e.g. the *Spirochaetes *cluster S. Although the secondary structure predictions mainly agreed with those of the other LytR-CpsA-Psr members, there was a very weak conservation of amino acids (data not shown), possibly indicating a different function.

### Confirmation of membrane topology

The predicted membrane topology of the LytR-CpsA-Psr proteins was verified by PhoA fusions using the staphylococcal protein MsrR as a representative of the protein family. The amino acid sequence of MsrR is identical in twelve of the 14 sequenced *S. aureus *strains available on the NCBI website [16] and there is only one amino acid difference in strains Mu50 and Mu3, which both have lysine at position 146 instead of glutamic acid (Additional files 5 and 6). For the following analyses, the sequence represented by *S. aureus *strain N315 was employed (see also Additional file 4).

#### PhoA fusions and activity assay

**Figure 7:**
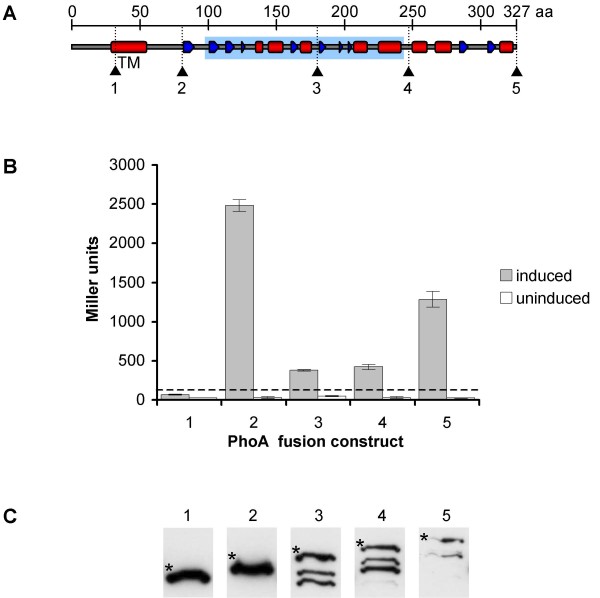
**Confirmation of membrane topology of MsrR**. (A) Schematic drawing of MsrR showing the predicted secondary structure (red barrel, α-helix; blue arrow, β-sheet) and transmembrane helix (TM). Black arrows indicate the positions where MsrR was fused to PhoA yielding fusion constructs number 1 (Δ295, deletion of 295 aa from the C-terminus), 2 (Δ246), 3 (Δ147), 4 (Δ80), and 5 (full length). (B) PhoA activity of fusion constructs after induction with arabinose as well as of uninduced control samples. The average and standard deviations of the results obtained using three different clones of each fusion construct are shown. The dashed line indicates a threshold of 120 Miller Units, which equals twice the highest background activity measured. (C) Detection of PhoA fusion constructs by Western blot hybridization using α-PhoA antibodies. For all fusion constructs, bands corresponding to the expected size were detected (marked by an asterisk): 52.6 kDa (1), 58.0 kDa (2), 69.3 kDa (3), 76.9 kDa (4), and 85.5 kDa (5). In the lanes containing constructs number 3–5, several smaller bands were present, which most probably represent degradation products.

Five different PhoA fusion proteins were constructed, with MsrR lacking either 0, 80, 147, 246, or 295 amino acids from the C-terminus (full length, Δ80, Δ147, Δ246, and Δ295) (Figure 7A), and were expressed in the *phoA*^- ^*E. coli *strain CC118. Correct folding of PhoA relies on the formation of disulfide bonds and occurs in the periplasm only. Therefore, PhoA activity can only be measured if the fusion protein spans the membrane in a suitable orientation. The fusions of PhoA to the full length protein and to MsrR truncated by 80, 147, and 246 amino acids from the C-terminus led to significant PhoA activity, indicating that PhoA had been translocated to the periplasmic space (Figure 7B), however, there were large variations in activity between the different fusion constructs. The highest activity was reached when PhoA was fused to MsrR shortly after the transmembrane domain (Δ246), whereas the fusions towards the C-terminus (Δ147, Δ80, and full length) showed lower activities. Western blot hybridization using α-PhoA antibodies revealed partial degradation of the Δ147, Δ80, and full length fusion constructs, which likely accounts for the observed differences in PhoA activity (Figure 7C). In contrast, one strong, single band of the expected size was detected for the Δ246 as well as for the Δ295 fusion constructs. The Δ295 fusion lacks the transmembrane domain and did not yield PhoA activity above the threshold value, indicating that the fusion construct remained in the cytoplasm.

These findings confirmed the predicted arrangement of the LytR-CpsA-Psr protein MsrR in the membrane: The short N-terminal tail is located inside of the cell and is followed by one transmembrane helix and an extracellular domain carrying the LytR-CpsA-Psr domain. Most members of the LytR-CpsA-Psr protein family show very similar predicted architectures as MsrR.

## Conclusion

Members of the LytR-CpsA-Psr family are known to influence clinically important attributes of various pathogens [2,4,5]. The LytR-CpsA-Psr domain was found to be restricted to the kingdom of bacteria. Being present in hyperthermophilic organisms of evolutionary early branches, the LytR-CpsA-Psr proteins appear to constitute an ancient protein family. All Gram-positive organisms were found to possess LytR-CpsA-Psr proteins, except for the *Mollicutes *that seem to have lost this protein family in the course of diversification. In contrast, the LytR-CpsA-Psr domain was only observed in a minority of the Gram-negatives, and it is conceivable that the LytR-CpsA-Psr proteins were lost in *Proteobacteria *and *Chlamydiae *as well as in most of the *Bacteroidetes *during evolution. LytR-CpsA-Psr proteins are shared by the most diverse procaryotic phyla, including the thermophiles of the first bacterial branches, and the genes encoding LytR-CpsA-Psr proteins often belong to the core genome. Although these proteins do not appear to be essential, they presumably confer a selective advantage to their carriers, as becomes apparent in vitro when mutant cells are challenged with antibiotic, acidic, or oxidative stress [4,5].

The finding that various members of this protein family are part of the cell wall stress stimulon in different organisms [30,33-38] might further suggest a role in ensuring cell envelope integrity. Nevertheless, the specific functions of the LytR-CpsA-Psr proteins are probably more diverse than originally anticipated, as indicated by the results of the cluster analysis, explaining discrepancies in phenotypes of knock out mutants [1-4,7].

Our analyses of the occurrence and distribution of the LytR-CpsA-Psr proteins as well as the determination of putative structural elements and conserved amino acids of the family-specific domain will provide a guideline for the design of future experiments to elucidate structure-function relationships.

## Methods

### Sequence collection

As of August 20, 2008, accession numbers of LytR-CpsA-Psr protein family members included in the InterPro database (InterPro entry IPR004474), a consortium of the member databases PROSITE, Pfam, Prints, ProDom, SMART, and TIGRFAMs [12], were obtained. Homologues sequences not present in InterPro were identified by PSI-BLAST [13] against the NCBI non-redundant protein sequence database (nr) using MsrR of *S. aureus *strain N315 [UniProt:Q99Q02] as a query and an E-value threshold of 10^-4^. Amino acid sequences were retrieved from UniProt [49] or from NCBI [16].

### Cluster analysis

To identify closely related sequences within the LytR-CpsA-Psr members, CLANS (CLuster ANalyis of Sequences) was used [24]. For two-dimensional visualization of pairwise sequence similarities, based on P-values of high-scoring segment pairs (HSPs) resulting from a BLAST all against all search, the P-value cut off was set to 10^-35 ^and the attraction (attract) and repulsion (repuls) exponents were set to 2. Network-based clustering was applied to assist designation of clusters.

### Structure analysis

Sequence compositions and theoretical pI were analyzed using ProtParam [50]. Transmembrane regions were predicted using TMHMM [51], the "DAS"-Transmembrane Prediction server [52], TMpred [53], and HMMTOP [54]. Secondary structures were predicted using SsPro [55], PSIPRED [56], and Jpred [57].

### Amino acid sequence alignments

Amino acid sequences were aligned using MUSCLE version 3.6 [58] and protein identity matrices of the MUSCLE alignments were calculated using ClustalX 2.1 [59].

### Phylogenetic trees

Phylogenetic trees were constructed using programs of the PHYLIP 3.67 package [60]. For construction of a species tree, aligned 16S rRNA genes were downloaded from the ribosomal RNA database RDP-II [61]. Misaligned characters were manually edited using SeaView version 2.2 [62]. The C- and N-termini were trimmed and gap-only sites were deleted from the alignment. A distance matrix, computed with DNADIST using the F84 model of nucleotide substitution, was used for construction of a distance tree with FITCH (Fitch-Margoliash criterion) allowing global rearrangements and randomizing the input order of the sequences 10 times. For construction of protein sequence trees, bootstrap resampling was performed using SEQBOOT. Consensus distance trees were generated using PROTDIST with the Jones-Taylor-Thornton substitution matrix, NEIGHBOR, and CONSENSE. Phylogenetic trees were drawn with Dendroscope version 1.2.4 [63].

### Bacterial strains and culture conditions

Strains and plasmids used in this study are listed in Table 1. Bacteria were maintained at 37°C on Luria Bertani (LB) (Difco Laboratories, Detroit, MI, USA) agar plates supplemented with 100 μg/ml ampicillin or on sheep-blood agar plates.

**Table 1:** Strains and plasmids

Strain or plasmid	Relevant genotype or phenotype^a)^	Reference or source
**Strains**		

*S. aureus*		
Newman	Clinical isolate (ATCC 25904)	[66]
*E. coli*		
DH5α	*supE*44 Δ*lacU*169(φ 80 *lacZ*ΔM15) *hsdR*17 *recA*1 *endA*1 *gyrA*96 *thi*-1 *rel*A1	Invitrogen
CC118	Δ(ara-leu)7679 Δ*lacX*74 Δ*phoA*20 *galE galK thi rpsE rpoB argE*(am) *recA*1	[67]

**Plasmids**		

pHA-1	*E. coli *plasmid containing an arabinose inducible promoter 5' of a signal sequence-less *phoA *reporter gene, Am^r^	[68]

^a)^Am^r^, Ampicillin resistance marker

### PhoA fusion constructs

PCR products covering different lengths of *msrR *of *S. aureus *strain Newman were generated using the forward primer 5'-GCGCCTCGAGATGGATAAAGAAACTAATGA-3' and the following reverse primers: 5'-GCGCGGTACCTCTTCATCTAAAAAGTCTTT-3' (full length), 5'-GCGCGGTACCGTTCTAAAATTAACCATTTC-3' (Deletion of 80 aa from the C-terminus, Δ80), 5'-GCGCGGTACCTCAGGCATTAATTCATCAA-3 (Δ147), 5'-GCGCGGTACCCCATCATTTTTTACTGGTCC-3' (Δ246), and 5'-GCGCGGTACCAATTTCCTAATTTTCTTCTT-3' (Δ295). Recognition sites of restriction endonucleases are underlined. The amplicons were digested using XhoI and KpnI and inserted into pHA-1 downstream of the *araB *arabinose-inducible promoter and upstream of the *phoA *gene lacking both the 5' segment coding for the signal sequence and the first five residues of the mature protein. The resulting plasmids were transferred into the *phoA*^- ^*E. coli *strain CC118.

### Expression of PhoA fusion proteins and PhoA activity assay

Overnight cultures of *E. coli *strain CC118 harboring the *msrR-phoA *fusion constructs of interest were diluted 1:100 in LB broth containing 100 μg/ml ampicillin and grown to an optical density at 600 nm of 0.5. Bacterial cultures were then divided into halves: in one half, protein expression was induced with 0.2% arabinose, while the other half was left untreated. After growth for an additional hour, 1 ml of induced and non-induced cells, respectively, were harvested for determination of PhoA activity by a *p*-nitrophenyl phosphate (*p*NPP) (Sigma-Aldrich) cleavage assay [64]. In addition, 200 μl of each cell suspension were collected for verification of PhoA fusion protein expression by Western blot using an α-PhoA antibody conjugated to horseradish peroxidase (HRP) (Abcam Ltd., UK).

### Western blot analysis

Whole cells were heated for 5 min at 65°C in 5× SDS loading buffer supplemented with 0.05% *N*-lauroylsarcosyl and subjected to SDS-12%-PAGE. Separated proteins were transferred onto nitrocellulose membranes (Hybond-ECL; Amersham Biosciences), and for detection of PhoA, a HRP-conjugated α-PhoA antibody (Abcam) diluted 1:100'000 and the SuperSignal West Pico Chemiluminescent detection kit (Pierce) were used.

## Authors' contributions

JH carried out sequence-based analyses, performed PhoA assays and Western blots, and drafted the manuscript. LL constructed PhoA fusion plasmids. BBB participated in the design and coordination of the study and contributed to the writing of the manuscript. PSM participated in the design of the study and contributed to the interpretation of the results and writing of the manuscript. All authors read and approved the final manuscript.

## Supplementary Material

Additional file 1 **Results of CLANS clustering analysis.** CLANS clusters of the LytR-CpsA-Psr proteins based on pair-wise sequence similarities resulting from a BLAST all against all search using a P-value cut off of 10^-35^. The clusters were named using the first character(s) of the respective bacterial phylum and were numbered in case there were multiple clusters of the same phylum. The NCBI GI numbers and UniProt/SwissProt accessions are given. A, *Actinobacteria*; Ch, *Chloroflexi*; Cy,*Cyanobacteria*; D, *Deinococcus-Thermus*; F, *Firmicutes*; S, *Spirochaetes*; T, *Thermotogae*. Click here for file

Additional file 2 **LytR-CpsA-Psr proteins used in phylogenetic analyses.** Table showing the LytR-CpsA-Psr family members identified in the 30 selected, completely sequenced bacterial strains. Click here for file

Additional file 3 **Neighbour-joining tree of LytR-CpsA-Psr proteins.** A phylogenetic tree was constructed based on a MUSCLE alignment of the full length sequences. Bootstrap values only support the branching order towards the terminal nodes and deeper branches have to be considered unresolved (red values). Using solely the LytR-CpsA-Psr domain for the initial alignment, changing parameters, e.g. for calculation of the distance matrix, or applying another tree construction algorithm did not improve the results (data not shown). The UniProt entry names are given, and the subtrees are colored using the same color scheme as in Figures 2 and 3 (blue, *Actinobacteria*; green, *Cyanobacteria*; yellow, *Chloroflexi*; turquoise, *Deinococcus-Thermus*; red, *Firmicutes*; petrol, *Thermotogae*). Brackets indicate the corresponding CLANS clusters. Click here for file

Additional file 4 **Primary sequence analyses of LytR-CpsA-Psr proteins and results of transmembrane region prediction.** The primary sequences of the full length proteins as well as of the LytR-CpsA-Psr domain as defined in the PFAM database (PF03816) were analyzed using ProtParam. Transmembrane segments were predicted using the web-based servers TMHMM, TMpred, Das, and HMMTOP. The first and the last residue of the predicted transmembrane domains are indicated. Click here for file

Additional file 5 **Percent identity matrices of LytR-CpsA-Psr protein sequences.** The sequence identifiers correspond to the UniProt entry names (see also Additional file 4). (A) Percent identity matrix of the full length sequences used for secondary structure prediction. The clusters as identified using CLANS are given. (B) Percent identity matrix of the extended LytR-CpsA-Psr domains of the sequences used for secondary structure prediction. For each sequence, the amino acid range is given in parentheses. (C) Percent identity matrix of the MsrR protein sequences in staphylococci. STAS1, *S. saprophyticus *ATCC15305; STAAN, *S. aureus *N315; STAAM, *S. aureus *Mu50; STAES, *S. epidermidis *ATCC12228; STAHJ, *S. haemolyticus *JCSC1435. Click here for file

Additional file 6 **Sequence comparison of MsrR in staphylococci.** The lysine (K) residue at position 146 of MsrR of *S. aureus *Mu50 and Mu3 is indicated by a red box. The LytR-CpsA-Psr domain as defined in the PFAM database (PF03816) is shaded in grey, and conserved residues as determined in Figure 6 are highlighted in blue. Residues of the predicted transmembrane regions are printed in red. Sequences are labelled using UniProt entry names. STAS1, *S. saprophyticus *ATCC15305; STAAN, *S. aureus *N315; STAAM, *S. aureus *Mu50; STAES, *S. epidermidis *ATCC12228; STAHJ, *S. haemolyticus *JCSC1435. Click here for file
