# Supplementary material for: Phylogenetic distribution and membrane topology of the LytR-CpsA-Psr protein family
Source: BMC Genomics. 2008 Dec 19;9:617. doi: 10.1186/1471-2164-9-617 (PMC2632651; doi:10.1186/1471-2164-9-617)

**Additional file 3 — Neighbour-joining tree of LytR-CpsA-Psr proteins**

A phylogenetic tree was constructed based on a MUSCLE alignment of the full length sequences. Bootstrap values only support the branching order towards the terminal nodes and deeper branches have to be considered unresolved (red values). Using solely the LytR-CpsA-Psr domain for the initial alignment, changing parameters, e.g. for calculation of the distance matrix, or applying another tree construction algorithm did not improve the results (data not shown). The UniProt entry names are given, and the subtrees are colored using the same color scheme as in Figures 2 and 3 (blue, *Actinobacteria*; green, *Cyanobacteria*; yellow, *Chloroflexi*; turquoise, *Deinococcus-Thermus*; red, *Firmicutes*; petrol, *Thermotogae*). Brackets indicate the corresponding CLANS clusters.

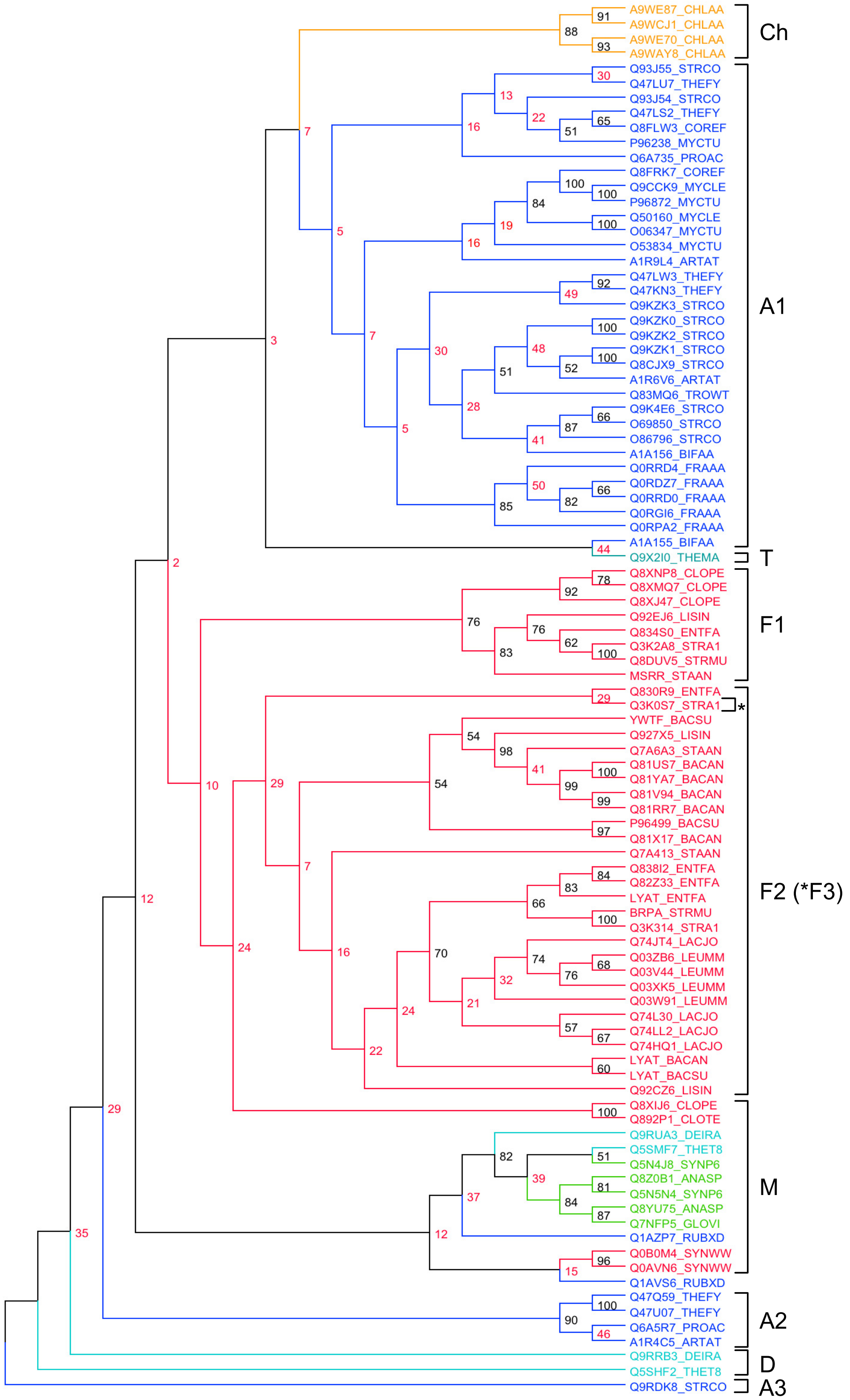

Supplement: Additional file 3 — Neighbour-joining tree of LytR-CpsA-Psr proteins. A phylogenetic tree was constructed based on a MUSCLE alignment of the full length sequences. Bootstrap values only support the branching order towards the terminal nodes and deeper branches have to be considered unresolved (red values). Using solely the LytR-CpsA-Psr domain for the initial alignment, changing parameters, e.g. for calculation of the distance matrix, or applying another tree construction algorithm did not improve the results (data not shown). The UniProt entry names are given, and the subtrees are colored using the same color scheme as in Figures 2 and 3 (blue, Actinobacteria; green, Cyanobacteria; yellow, Chloroflexi; turquoise, Deinococcus-Thermus; red, Firmicutes; petrol, Thermotogae). Brackets indicate the corresponding CLANS clusters. [file 1471-2164-9-617-S3.pdf]
