# Supplementary material for: Phylogenetic distribution and membrane topology of the LytR-CpsA-Psr protein family
Source: BMC Genomics. 2008 Dec 19;9:617. doi: 10.1186/1471-2164-9-617 (PMC2632651; doi:10.1186/1471-2164-9-617)
Supplement: Additional file 4 — Primary sequence analyses of LytR-CpsA-Psr proteins and results of transmembrane region prediction. The primary sequences of the full length proteins as well as of the LytR-CpsA-Psr domain as defined in the PFAM database (PF03816) were analyzed using ProtParam. Transmembrane segments were predicted using the web-based servers TMHMM, TMpred, Das, and HMMTOP. The first and the last residue of the predicted transmembrane domains are indicated. [file 1471-2164-9-617-S4.pdf]

Additional file 4 – Primary sequence analyses of LytR-CpsA-Psr proteins and results of transmembrane region prediction

The primary sequences of the full length proteins as well as of the LytR-CpsA-Psr domain as defined in the PFAM database (PF03816) were analyzed using ProtParam. Transmembrane segments were predicted using the web-based servers TMHMM, TMpred, Das, and HMMTOP. The first and the last residue of the predicted transmembrane domains are indicated.

UniProt entry name	LYAT_BACSU	Q3K0S7_STRA1	Q47828_ENTHR	MSRR_STAAN	Q7A6A3_STAAN	Q7A413_STAAN
Organism	*Bacillus subtilis*	*Streptococcus agalactiae*	*Enterococcus hirae*	*Staphylococcus aureus*	*Staphylococcus aureus*	*Staphylococcus aureus*
Strain	168	A909	ATCC 9790	N315	N315	N315
Gene	*lytR* (BSU3565)	*cpsX* (SAK_1262)	*psr*	*msrR* (SA1195)	SA0908	SA2103
UniProt accession	Q02115	Q3K0S7	Q47828	Q99Q02	Q7A6A3	Q7A413
Full length protein						
Molecular weight (Da)	34586.4	54131	32766.3	36970.7	45711.2	34737.5
Amino acids	306	485	293	327	405	315
K (+)	32	34	25	38	33	28
R (+)	15	16	10	17	15	14
K, R (+)	47	50	35	55	48	42
D (-)	25	23	26	22	36	24
E (-)	15	16	15	16	17	13
D, E (-)	40	39	41	38	53	37
(K, R) - (D, E)	7	11	-6	17	-5	5
Isoelectric point	9.18	9.32	5.17	9.66	6.02	9.11
LytR-CpsA-Psr domain	(83, 227)	(248, 395)	(68, 212)	(99, 244)	(86, 234)	(90, 236)
Amino acids	145	148	145	146	149	147
K (+)	9	8	13	15	12	10
R (+)	9	7	7	9	8	9
K, R (+)	18	15	20	24	20	19
D (-)	13	11	15	10	12	11
E (-)	5	5	7	9	7	7
D, E (-)	18	16	22	19	19	18
(K, R) - (D, E)	0	-1	-2	5	1	1
TM prediction						
TMHMM	13–35	19–41, 45–67, 74–96	7–29	32–54	7–28	15–34
TMpred	12–31	19–40, 52–70, 76–96	7–30	32–51	8–27	15–36
DAS	12–28	18–40, 50–70, 76–94	7–24	31–53	6–28	15–39
HMMTOP	11–34	20–39, 52–69, 76–93	7–24	32–51	7–26	15–39

Additional file 4 — continued

UniProt entry name	BRPA_STRMU	Q8FLW3_COREF	Q5N4J8_SYNP6	Q9X2I0_THEMA	Q9RUA3_DEIRA	A9WE87_CHLAA
Organism	*Streptococcus mutans*	*Corynebacterium efficiens*	*Synechococcus elongatus*	*Thermotoga maritima*	*Deinococcus radiodurans*	*Chloroflexus aurantiacus*
Strain	UA159	YS-314	PCC 6301	MSB8	R1	J-10-fl
Gene	*brpA* (SMU_410)	CE2746	syc0581_c	TM_1866	DR_1488	Caur_0499
UniProt accession	Q8DVR0	Q8FLW3	Q5N4J8	Q9X2I0	Q9RUA3	A9WE87
Full length protein						
Molecular weight (Da)	43909.2	45130	36897.4	52507	41734.6	51343.4
Amino acids	406	417	337	457	389	472
K (+)	34	1	8	35	11	3
R (+)	10	38	26	25	27	42
K, R (+)	44	39	34	60	38	45
D (-)	25	26	18	27	25	31
E (-)	18	17	11	32	11	20
D, E (-)	43	43	29	59	36	51
(K, R) - (D, E)	1	-4	5	1	2	-6
Isoelectric point	7.66	6.01	9.37	7.71	8.75	5.3
LytR-CpsA-Psr domain	(80, 238)	(197, 340)	(82, 231)	(117, 261)	(74, 223)	(111, 284)
Amino acids	159	144	150	145	150	174
K (+)	14	1	5	14	4	1
R (+)	6	9	16	6	13	16
K, R (+)	20	10	21	20	17	17
D (-)	13	10	11	10	11	17
E (-)	9	9	4	10	4	9
D, E (-)	22	19	15	20	15	26
(K, R) - (D, E)	-2	-9	6	0	2	-9
TM prediction						
TMHMM	7–29	123–145	12–31	65–84	7–29	44–66
TMpred	7–25	122–141	9–31	65–85	8–27	46–65
DAS	7–29	124–144	13–24	64–86	7–28	47–65*
HMMTOP	7–29	123–142	9–26	65–84	8–27	49–66**

* An additional TM segment was predicted from position 89 to 98.

** Two additional TM segments were predicted from position 83 to 101 and from 114 to 132.
