# Supplementary material for: Phylogenetic distribution and membrane topology of the LytR-CpsA-Psr protein family
Source: BMC Genomics. 2008 Dec 19;9:617. doi: 10.1186/1471-2164-9-617 (PMC2632651; doi:10.1186/1471-2164-9-617)
Supplement: Additional file 5 — Percent identity matrices of LytR-CpsA-Psr protein sequences. The sequence identifiers correspond to the UniProt entry names (see also Additional file 4). (A) Percent identity matrix of the full length sequences used for secondary structure prediction. The clusters as identified using CLANS are given. (B) Percent identity matrix of the extended LytR-CpsA-Psr domains of the sequences used for secondary structure prediction. For each sequence, the amino acid range is given in parentheses. (C) Percent identity matrix of the MsrR protein sequences in staphylococci. STAS1, S. saprophyticus ATCC15305; STAAN, S. aureus N315; STAAM, S. aureus Mu50; STAES, S. epidermidis ATCC12228; STAHJ, S. haemolyticus JCSC1435. [file 1471-2164-9-617-S5.pdf]

Additional file 5 — Percent identity matrices of LytR-CpsA-Psr protein sequences

(A) Percent identity matrix of the full length sequences used for secondary structure prediction. The clusters as identified using CLANS are given. The sequence identifiers correspond to the UniProt entry names according to Additional file 4.

	Cluster	Q9X2I0_THEMA (0-457)	MSRR_STAAN (0-327)	Q47828_ENTHR (0-293)	Q8FLW3_COREF (0-417)	Q5N4J8_SYNP6 (0-337)	Q9RUA3_DEIRA (0-389)	A9WE87_CHLAA (0-472)	Q7A6A3_STAAN (0-405)	Q3K0S7_STRA1 (0-485)	BRPA_STRMU (0-406)	Q7A413_STAAN (0-315)	LYAT_BACSU (0-306)
Q9X2I0_THEMA (0-457)	T	100	23	17	20	26	21	17	18	18	19	20	22
MSRR_STAAN (0-327)	F1	23	100	38	21	24	30	24	23	22	25	22	24
Q47828_ENTHR (0-293)	F1	17	38	100	26	24	27	29	26	23	23	27	28
Q8FLW3_COREF (0-417)	A1	20	21	26	100	23	30	28	23	19	20	23	23
Q5N4J8_SYNP6 (0-337)	M	26	24	24	23	100	31	29	21	19	22	24	21
Q9RUA3_DEIRA (0-389)	M	21	30	27	30	31	100	30	21	22	21	25	25
A9WE87_CHLAA (0-472)	Ch	17	24	29	28	29	30	100	21	18	19	24	26
Q7A6A3_STAAN (0-405)	F2	18	23	26	23	21	21	21	100	24	26	27	31
Q3K0S7_STRA1 (0-485)	F3	18	22	23	19	19	22	18	24	100	27	28	29
BRPA_STRMU (0-406)	F2	19	25	23	20	22	21	19	26	27	100	27	35
Q7A413_STAAN (0-315)	F2	20	22	27	23	24	25	24	27	28	27	100	39
LYAT_BACSU (0-306)	F2	22	24	28	23	21	25	26	31	29	35	39	100

(B) Percent identity matrix of the extended LytR-CpsA-Psr domains of the sequences used for secondary structure prediction. For each sequence, the amino acid range is given in parentheses. The sequence identifiers correspond to the UniProt entry names according to Additional file 4.

	Cluster	Q9X2I0_THEMA (87-612)	MSRR_STAAN (67-482)	Q47828_ENTHR (35-448)	Q8FLW3_COREF (151-572)	Q5N4J8_SYNP6 (42-492)	Q9RUA3_DEIRA (32-544)	A9WE87_CHLAA (83-627)	Q7A6A3_STAAN (46-560)	Q3K0S7_STRA1 (214-640)	BRPA_STRMU (43-561)	Q7A413_STAAN (53-470)	LYAT_BACSU (51-461)
Q9X2I0_THEMA (87-313)	T	100	29	22	26	32	28	26	26	22	26	26	26
MSRR_STAAN (67-298)	F1	29	100	41	27	26	32	28	27	26	27	26	29
Q47828_ENTHR (35-262)	F1	22	41	100	29	27	31	30	28	26	27	30	31
Q8FLW3_COREF (151-391)	A1	26	27	29	100	24	32	31	25	24	25	26	25
Q5N4J8_SYNP6 (42-278)	M	32	26	27	24	100	35	33	26	21	27	28	26
Q9RUA3_DEIRA (32-270)	M	28	32	31	32	35	100	32	27	25	28	28	28
A9WE87_CHLAA (83-345)	Ch	26	28	30	31	33	32	100	29	22	26	29	30
Q7A6A3_STAAN (46-282)	F2	26	27	28	25	26	27	29	100	27	31	29	35
Q3K0S7_STRA1 (214-445)	F3	22	26	26	24	21	25	22	27	100	30	32	34
BRPA_STRMU (43-287)	F2	26	27	27	25	27	28	26	31	30	100	32	39
Q7A413_STAAN (53-284)	F2	26	26	30	26	28	28	29	29	32	32	100	44
LYAT_BACSU (51-275)	F2	26	29	31	25	26	28	30	35	34	39	44	100

(C) Percent identity matrix of the MsrR protein sequences in staphylococci. Sequence identifiers correspond to UniProt entry names. STAS1, *S. saprophyticus* ATCC15305; STAAN, *S. aureus* N315; STAAM, *S. aureus* Mu50; STAES, *S. epidermidis* ATCC12228; STAHJ, *S. haemolyticus* JCSC1435.

	aa	*S. aureus* N315, MW2, MRSA252, MSSA475, COL, RF122, USA300, NCTC8325, JH, JH9, Newman	*S. aureus* Mu50, Mu3	*S. epidermidis* ATCC12228	*S. haemolyticus* JCSC1435	*S. saprophyticus* ATCC15305
S. aureus N315, MW2, MRSA252, MSSA475, COL, RF122, USA300, NCTC8325, JH, JH9, Newman	327	100	99	76	74	68
S. aureus Mu50, Mu3	327	99	100	75	73	68
S. epidermidis ATCC12228	329	76	75	100	79	72
S. haemolyticus JCSC1435	325	74	73	79	100	68
S. saprophyticus ATCC15305	327	68	68	72	68	100
