# Supplementary material for: Phylogenetic distribution and membrane topology of the LytR-CpsA-Psr protein family
Source: BMC Genomics. 2008 Dec 19;9:617. doi: 10.1186/1471-2164-9-617 (PMC2632651; doi:10.1186/1471-2164-9-617)
Supplement: Additional file 6 — Sequence comparison of MsrR in staphylococci. The lysine (K) residue at position 146 of MsrR of S. aureus Mu50 and Mu3 is indicated by a red box. The LytR-CpsA-Psr domain as defined in the PFAM database (PF03816) is shaded in grey, and conserved residues as determined in Figure 6 are highlighted in blue. Residues of the predicted transmembrane regions are printed in red. Sequences are labelled using UniProt entry names. STAS1, S. saprophyticus ATCC15305; STAAN, S. aureus N315; STAAM, S. aureus Mu50; STAES, S. epidermidis ATCC12228; STAHJ, S. haemolyticus JCSC1435. [file 1471-2164-9-617-S6.pdf]

Additional file 6 — Sequence comparison of MsrR in staphylococci

The lysine (K) residue at position 146 of MsrR of *S. aureus* Mu50 and Mu3 is indicated by a red box. The LytR-CpsA-Psr domain as defined in the PFAM database (PF03816) is shaded in grey, and conserved residues as determined in Figure 6 are highlighted in blue. Residues of the predicted transmembrane regions are printed in red. Sequences are labelled using UniProt entry names.

STAS1, *S. saprophyticus* ATCC15305; STAAN, *S. aureus* N315; STAAM, *S. aureus* Mu50; STAES, *S. epidermidis* ATCC12228; STAHJ, *S. haemolyticus* JCSC1435.

```
                     * ::.    .  . *    . . :* :**::****:*:*: :*:*   : * .* *.**::**:.*.* :* *:*** :*
Q49XG2_STAS1         MNEDKL--RSSKNTQGNTNPPPMRRGKKKRFRKLPFIILIGIIILAIIIGYSVHGYNSGIKYAKDHGKTLKQHKFNGAVK   78
MSRR_STAAN           MDKETN--DNEYRRQSEHRTSAPKRKKKKKIRKLPIILLIVVILLIALVVYIVHSYNSGVEYAKKHAKDVKVHQFNGPVK   78
MSRR_STAAM           MDKETN--DNEYRRQSEHRTSAPKRKKKKKIRKLPIILLIVVILLIALVVYIVHSYNSGVEYAKKHAKDVKVHQFNGPVK   78
Q8CPB8_STAES         MTEQKDIKETEYRRQKGTTSTPSRRRNKKRMRKLPFIILVILIILISIIVYITHQYNSGMKYAKEHAKDVKVHKFNGNMK   80
Q4L669_STAHJ         MTEEKD--KTVYRRQADAQT-PSKRRNKKKLRKLPFIILIILILLIAAIIYIVHGYRSGVNYAEKHAKDIKVHKFNGPVK   77

                     *****:::****** .** **:****:.***:::**************:***  ::***:**::***:***:**.****:
Q49XG2_STAS1         NDGKITVMVLGADKEQGGISRTDSIMIAQYDYIHKKMKMMSVMRDIYADIPGNGQHKINSAYSIGGPELLRQTLKKNLGI 158
MSRR_STAAN           NDGKISILVLGADKAQGGQSRTDSIMVVQYDFINKKMKMMSVMRDIYADIPGYGKHKINSAYALGGPELLRKTLDKNLGI 158
MSRR_STAAM           NDGKISILVLGADKAQGGQSRTDSIMVVQYDFINKKMKMMSVMRDIYADIPGYGKHKINSAYALGGPKLLRKTLDKNLGI 158
Q8CPB8_STAES         NDGKISVLVLGADKAQGGKSRTDSIMIVQYDYVHKKMKMMSVMRDIYADIPGYDKYKINAAYSLGGPELLRKTLNKNLGV 160
Q4L669_STAHJ         NDGKISVLVLGADKASGGKSRSDSIMVVQYDYIHKKMKMMSVMRDIYAEIPGYQNYKINAAYSLGGPELLRKTLNKNLGI 157

                     :*****::*********** *:***::******:*********:*:*************** ***************:*
Q49XG2_STAS1         DPEYYAILDFTGFEKMIDELEPNGVPIDVEKDMSKNIGVSLKKGHHRLNGKELLGYARFRHDPEGDFGRVRRQQQVMQSL 238
MSRR_STAAN           NPEYYAVVDFTGFEKMIDELMPEGVPINVEKDMSKNIGVSLKKGNHRLNGKELLGYARFRHDPEGDFGRVRRQQQVMQTL 238
MSRR_STAAM           NPEYYAVVDFTGFEKMIDELMPEGVPINVEKDMSKNIGVSLKKGNHRLNGKELLGYARFRHDPEGDFGRVRRQQQVMQTL 238
Q8CPB8_STAES         NPEYYAVVDFTGFEKMIDELQPNGVPIDVEKDMSENIGVSLKKGHHKLNGKELLGYARFRHDPEGDFGRVRRQQQVMQTL 240
Q4L669_STAHJ         NPEYYAVIDFTGFEKMIDELEPNGVPMDVEKDMSENIGVSLKKGHHRLNGKELLGYARFRHDEEGDFGRVRRQQQVMQTL 237

                     *:*:..  :: ************:**.:*:* ::*:*:.:****:*.* *:*:**:.:*::::*  *****:*:. .**
Q49XG2_STAS1         KQELASLDTLPKLPKVAGILRGYLNTNMPDSVLIQSGLNFGIRGDKKVVSVTLPIKGTYQDIQTPMDGSALEIDKAKNKA 318
MSRR_STAAN           KKEMVNFRTVVKLPKVAGILRGYVNTNIPDSGIFQTGLSFGIRGEKDVKSLTVPIKNSYEDVNTNTDGSALQINKNTNKQ 318
MSRR_STAAM           KKEMVNFRTVVKLPKVAGILRGYVNTNIPDSGIFQTGLSFGIRGEKDVKSLTVPIKNSYEDVNTNTDGSALQINKNTNKQ 318
Q8CPB8_STAES         KQELVNFNTVAKLPKVAGILRGYVNTNMPNSAIFQTGISFGIRGDKDVQSLTVPIKGSYQDINTNNDGSALQIDSEKNKQ 320
Q4L669_STAHJ         KKELVTPSSVIKLPKVAGILRGYINTSMPNSAIFQTGISYGIRGDKNVESLTVPINNSYQNINTNNDGSALEIDKEKNKK 317

                      :*.*:::
Q49XG2_STAS1         EVKKFLEED 327
MSRR_STAAN           AIKDFLDED 327
MSRR_STAAM           AIKDFLDED 327
Q8CPB8_STAES         AIKNFFEDN 329
Q4L669_STAHJ         AVKEFLNN- 325
```
